# Multi-class deep learning architecture for classifying lung diseases from chest X-Ray and CT images

**DOI:** 10.1038/s41598-023-46147-3

**Published:** 2023-11-08

**Authors:** Mona Hmoud Al-Sheikh, Omran Al Dandan, Ahmad Sami Al-Shamayleh, Hamid A. Jalab, Rabha W. Ibrahim

**Affiliations:** 1 Physiology Department, College of Medicine, Imam Abdulrahman Bin Faisal University, 34212 Dammam, Saudi Arabia; 2 Department of Radiology, College of Medicine, Imam Abdulrahman Bin Faisal University, 34212 Dammam, Saudi Arabia; 3 Department of Data Science and Artificial Intelligence, Faculty of Information Technology, Al-Ahliyya Amman University, Al-Salt, Amman, 19328 Jordan; 4 Information and Communication Technology Research Group, Scientific Research Center, Al-Ayen University, Nile Street, 64001 Thi-Qar, Iraq; 5 Department of Mathematics, Mathematics Research Center, Near East University, Near East Boulevard, PC: 99138 Nicosia/Mersin 10, Turkey; 6 Department of Computer Science and Mathematics, Lebanese American University, Beirut, 1102 2801 Lebanon

**Keywords:** Computational biology and bioinformatics, Mathematics and computing

## Abstract

Medical imaging is considered a suitable alternative testing method for the detection of lung diseases. Many researchers have been working to develop various detection methods that have aided in the prevention of lung diseases. To better understand the condition of the lung disease infection, chest X-Ray and CT scans are utilized to check the disease’s spread throughout the lungs. This study proposes an automated system for the detection multi lung diseases in X-Ray and CT scans. A customized convolutional neural network (CNN) and two pre-trained deep learning models with a new image enhancement model are proposed for image classification. The proposed lung disease detection comprises two main steps: pre-processing, and deep learning classification. The new image enhancement algorithm is developed in the pre-processing step using k-symbol Lerch transcendent functions model which enhancement images based on image pixel probability. While, in the classification step, the customized CNN architecture and two pre-trained CNN models Alex Net, and VGG16Net are developed. The proposed approach was tested on publicly available image datasets (CT, and X-Ray image dataset), and the results showed classification accuracy, sensitivity, and specificity of 98.60%, 98.40%, and 98.50% for the X-Ray image dataset, respectively, and 98.80%, 98.50%, 98.40% for the CT scans dataset, respectively. Overall, the obtained results highlight the advantages of the image enhancement model as a first step in processing.

## Introduction

Research studies on the identification and treatment of lung disease infection have gotten great interest around the world. Most scientific studies revealed that different lung disease symptoms can be seen in X-Ray and CT images of the lungs. The wide availability of X-Ray and CT made these two imaging modalities a suitable way for early detection of lung disease infection. To accelerate the image analysis, many efforts have been done so far to implement artificial intelligence (AI) for improving the healthcare system^1^. The major achievements of deep learning (DL) approaches in detecting certain irregularities in medical images, motivated researchers to learn more about deep CNN architectures for lung disease classification in both X-Ray and CT scans^2^. There are two types of DL models: non-trained and pre-trained DL. Non-trained DL models must be trained first from scratch and require many images. While, the pre-trained DL, on the other hand, have already been trained using public image datasets. Because higher-order, semantic features are extracted from pre-trained models, the performance of such models is superior in most domains when compared to other image classification methods. In this study, two pre-trained CNN models, Alex Net and VGG16Net, are developed for discriminating three lung diseases: COVID-19, pneumonia, and normal (healthy) lung both X-Ray and CT scans. This study also proposes a pre-processing step using a novel k-symbol Lerch transcendent functions model.

## Background of the study

This section reviews the studies on methods for classifying lung diseases, which includes a variety of viewpoints and methodologies.

### Image enhancement

Image enhancement involves performing adjustments to digital images to make them more suitable for image analysis. The goal of image enhancement is to improve the brightness, contrast, and sharpness of the input image. The image enhancement technique is applied as a pre-processing step before the image classification task^3^. Numerous attempts have been made to automatically improve images related to lung images using different approaches. The study of Navaneetha et al^4^ introduced sunflower optimization for medical image enhancement. Utilizing the modified median filter, the input medical images are initially denoised. The pixel intensity of the image is then used to improve these denoised images. The potential limitation of this work is the parameter tuning like population size, maximum iterations, and specific parameters related to the modified version of the algorithm. Another kidney image enhancement approach based on image entropy was proposed by Al-Shamasneh et al^5^. With low contrast kidney MRI scans, this method produced good outcomes. Moreover, the study of Jalab et al^6^ introduced another image enhancement method based on fractional calculus to enhance fine features dynamically in response to image contents. This approach combines fractional integral and entropy to solve the problem of image enhancement. In the new approach based on fractional calculus, Ibrahim et al.^7^ introduced a novel fractional partial differential class to enhance low contrast images of the brain and lungs. However, this method developed to enhance low-resolution images. A similar study using new fractional Rényi entropy model was proposed by Jalab et al.^8^ to enhance the kidney MRI. The fractional calculus operators have been applied as a novel method for improving images^7–9^. Regardless of how these studies perform as image enhancing models, an improvement is still conceivable.

### Deep learning for classifying lung diseases

Recently, many studies have been conducted with the help of different AI-based methods^10,11^. Numerous studies produced encouraging findings for using AI tools in classifying of lung diseases depending on certain features extracted from lung images. Deep learning (DL) which is a part of machine learning that uses artificial neural networks. DL is used widely in image classification, and recognition. Classifying of lung diseases is a challenging problem that requires specialized techniques. Deep learning approaches usually use a "convolutional neural network" (CNN). In Several image processing tasks, deep learning has demonstrated promising results, including classifying of lung diseases in recent years. To distinguish between infected and healthy lung tissue, the study by Saood and Hatem^12^ proposed deep learning networks, SegNet and U-NET. The findings in this study helped with the objective diagnosis of COVID-19 disease using lung CT scans. The results demonstrate SegNet’s superior ability to accurately classify infected and non-infected tissues with a 95% mean accuracy rate. Likewise, in Pereira et al.^13^ a chest X-Ray multi-class classification method has been proposed using texture as the key visual properties of X-Ray images. However, due to the long processing time, such a feature retrieval method inevitably increases the complexity of the calculation. In the same approach, Ismael and Şengür^14^, proposed a pre-trained CNN model for lung disease detection in X-Ray images. The obtained accuracy was 94.7%. However, the proposed system was based on the augmentation of the image database. While Öztürk et al.^15^ proposed X-Ray and CT image classification for lung diseases using the combination of standard feature extraction algorithms. However, the 98% achieved accuracy was due to the small testing images. A similar study by Hasan et al.^1^ used a combination of deep learning and handcrafted features to classify COVID-19 CT scans. Furthermore, Li et al.^16^ developed a volumetric CT scans image classification to detect cases of lung diseases. The reported specificity was 96%, and the sensitivity was 90%. Another model by Maghdid et al.^17^ proposed a pre-trained CNN model with an accuracy of 94.1% for classifying X-Ray images as normal or containing pneumonia. However, the problem, is that it is dependent on a prior model, is susceptible to overfitting, and cannot extract only the affected patterns. Furthermore, the accuracy of this model remains low, despite being higher than in previous research. Furthermore, a CNN models were employed in the Bhimavarapu et al.^18^ study to identify pneumonia and COVID-19. The collected dataset was run through each CNN model to extract the features, which were then applied as input to the classification models. This study demonstrated that the performance of the classification process was improved by obtaining deep features from the CNN models' common layers.

Research gaps concerning CT and X-Ray chest scans for lung diseases.

1. Degradation of the imaging quality due to image artifacts, patient movement, or technical errors makes disease detection more difficult.
2. While combining modalities, such as CT and X-Ray, or predicting multiple conditions or attributes at once may lead to improved performance, it also comes with its own set of difficulties.
3. Even though transfer learning has been used, further study should be done to improve performance on lung disease classification tasks by utilizing models trained on non-medical datasets or on other medical tasks. Further investigation in these fields may lead to enhanced diagnostic instruments and better patient outcomes.

The diagnostic performance is enhanced by classification, and the evaluation metrics have significantly improved. To mitigate all the above-mentioned drawbacks, this study proposed an image enhancement algorithm based on k-symbol Lerch transcendent functions model with a customized CNN and two pre-trained deep learning models for classification of three different lung diseases using publicly available image datasets. The following are the study contributions:

1. A novel image enhancement algorithm based on the k-symbol Lerch transcendent functions to enhance the images as well as to produce an efficient feature for CNNs models.
2. A customized CNN model with four layers has been proposed for classification of three different lung diseases in X-Ray and CT scans.
3. Two pre-trained deep learning models have been proposed as well for classification of three different lung diseases in X-Ray and CT scans.
4. Several experiments are carried out to show that the suggested approach performs better than state-of-the-art methods for classification of three different lung diseases.

## Materials and methods

**Figure 1:**
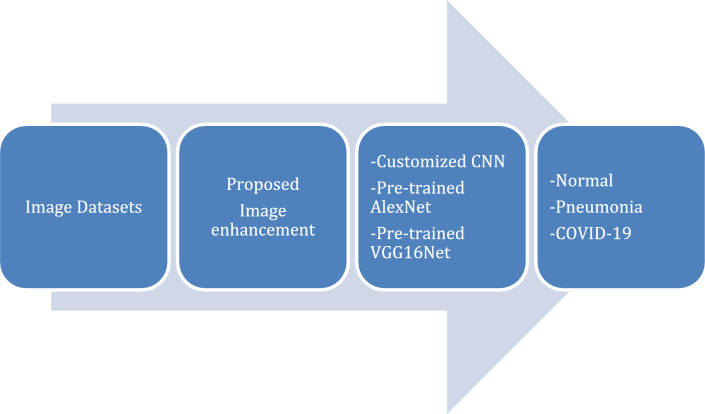
The flow of the algorithm.

The methodology used in the study is depicted in Fig. 1 as a block diagram. The study examined two types of lung images: CT scans of the lungs and X-Rays of the chest from publicly available datasets. The three main approaches in this study are presented: the proposed image enhancement model, the proposed customized CNN model, and the two tuned-pre-trained deep learning models for three different lung disease classifications.

### Proposed image enhancement model

Medical imaging systems, including CT and X-Ray technology have revolutionized healthcare and diagnostics by allowing medical professionals to make non-invasive diagnoses of diseases. However, occasionally, there may be artifacts, noise, or low resolution in medical images, which can cause difficulty in interpretation or misdiagnosis. Therefore, minimizing the effects of these causes before any image processing is crucial to reduce the likelihood of misclassifying pixels by CNN models. We investigate a new image enhancement model based on non-linear functions, which is motivated by the fact that non-linear functions can solve complex issues like non-linear complexities.

There are some benefits of using non-linear functions in image enhancement including capturing complex textures, and edge-preserving. Lerch transcendent functions (LTFs) are type of non-linear enhancement that can deal with the natural non-linearities in images, like noise and lighting changes, better than other non-linear enhancement techniques. Compared to linear methods, this might produce results for image enhancement that are more realistic. The Lerch transcendent provides an overall structure for the investigation of a variety of specific instances of special functions in number theory, and specific number theoretical constants. Additionally, fractional calculus can capture image detail by enhancing image contrast intensities. By applying the k-fractional symbol in Lerch transcendent function, improve the image enhancement by modifying the image's pixel values to enhance the visual quality of the image. The detail of the proposed model of k-fractional symbol in the Lerch transcendent function (K-LTF) for image enhancement is described in this subsection.

Lerch transcendent functions (LTFs) are very interesting since significant transcendental functions in their analytic extensions. Remember that the following series is used to define the Lerch transcendent function (LTF)^19^:

1 $$L\left( {p,\alpha ,\beta } \right) = { }\mathop \sum \limits_{n = 0}^{\infty } \frac{{p^{n} }}{{\left( {\alpha + n} \right)^{\beta } }},$$

where p, α and β are parameters. LTF has been modified using different types of power series. For example, it generalized associating to the Zeta function to obtain the Lerch-Zeta transcendent function^20^, as well as the Fourier series to get the Lerch-Fourier transcendent function^21^.

The Pochhammer k-symbol and the k-gamma function were first developed by Diaz and Pariguan^22^. The Pochhammer *k*-symbol (*p*)_*n,k*_ is defined as

2 $$\left( p \right)_{n,k} { } = {\text{ p}}\left( {{\text{p}} + {\text{k}}} \right)\left( {{\text{p}} + 2{\text{k}}} \right) \ldots \left( {{\text{p}} + \left( {{\text{n}} - 1} \right){\text{k}}} \right)$$

By using the k-fractional symbol notion in Eq. (1), we have the k-fractional symbol Lerch transcendent function (K-LTF)

3 $$L_{k} \left( {p,\alpha ,\beta } \right) = { }\mathop \sum \limits_{n = 0}^{\infty } \frac{{\left( p \right)_{n,k} }}{{\left( {\alpha + n} \right)^{\beta } }}$$

**Figure 2:**
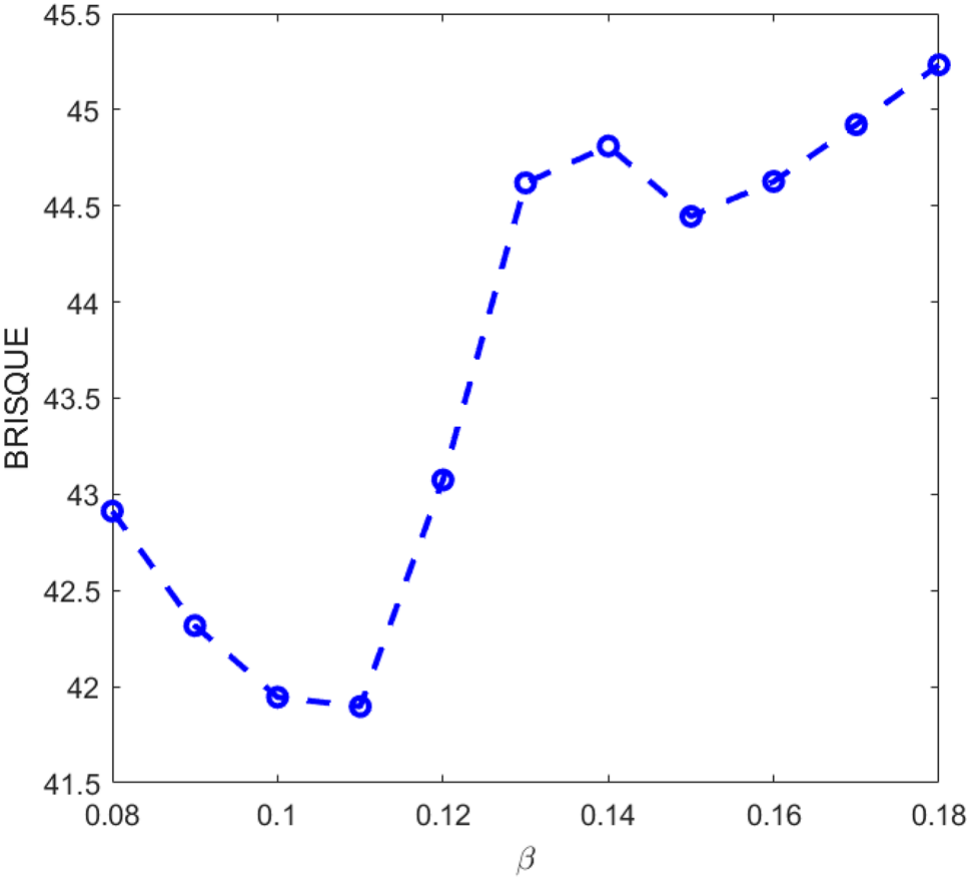
The average of BRISQUE with respect to different values of β.

For 2-Dimension image of size (*i,j*), since $\frac{1}{{\left( \alpha \right)^{\beta } }}$ > $\frac{1}{{\left( {\alpha + \left( {i,j} \right)} \right)^{\beta } }}$, we have:

4 $$L_{k} \left( {p,\alpha ,\beta } \right) = \mathop \sum \limits_{i = 1}^{r} \mathop \sum \limits_{j = 1}^{q} \frac{{\left( p \right)_{{\left( {i,j} \right),k}} }}{{\left( \alpha \right)^{\beta } }}$$

where α,and β are the fractional parameters, p represents the pixel probability of the input image and k = 1,2,…n. The proposed K-LTF-based image enhancement model is described in Eq. (4). The proposed K-LTF image enhancement model is intended to improve the image pixels by improving images with minor gray-level changes based on the probability of each pixel. By estimating the enhanced values for each pixel based on the pixel probability details of the image pixels, the K-LTF image enhancement model can improve low contrast intensities. To choose the right value of fractional parameter β, we considered α = 0.5. The standard quality measure, BRISQUE “Blind Reference less Image Spatial Quality Evaluator” has been chosen to evaluate the enhanced image quality^23^. The low value of BRISQUE represents the best image quality. The parameter β is crucial for better enhancement results. As described in Eq. (5), the parameters α, and β are the power parameters of the proposed model. The value of β has been chosen, as shown in Fig. 2. The best value for β is 0.11, in which the proposed method achieved the best BRISQUE score, which is the lowest.

**Figure 3:**
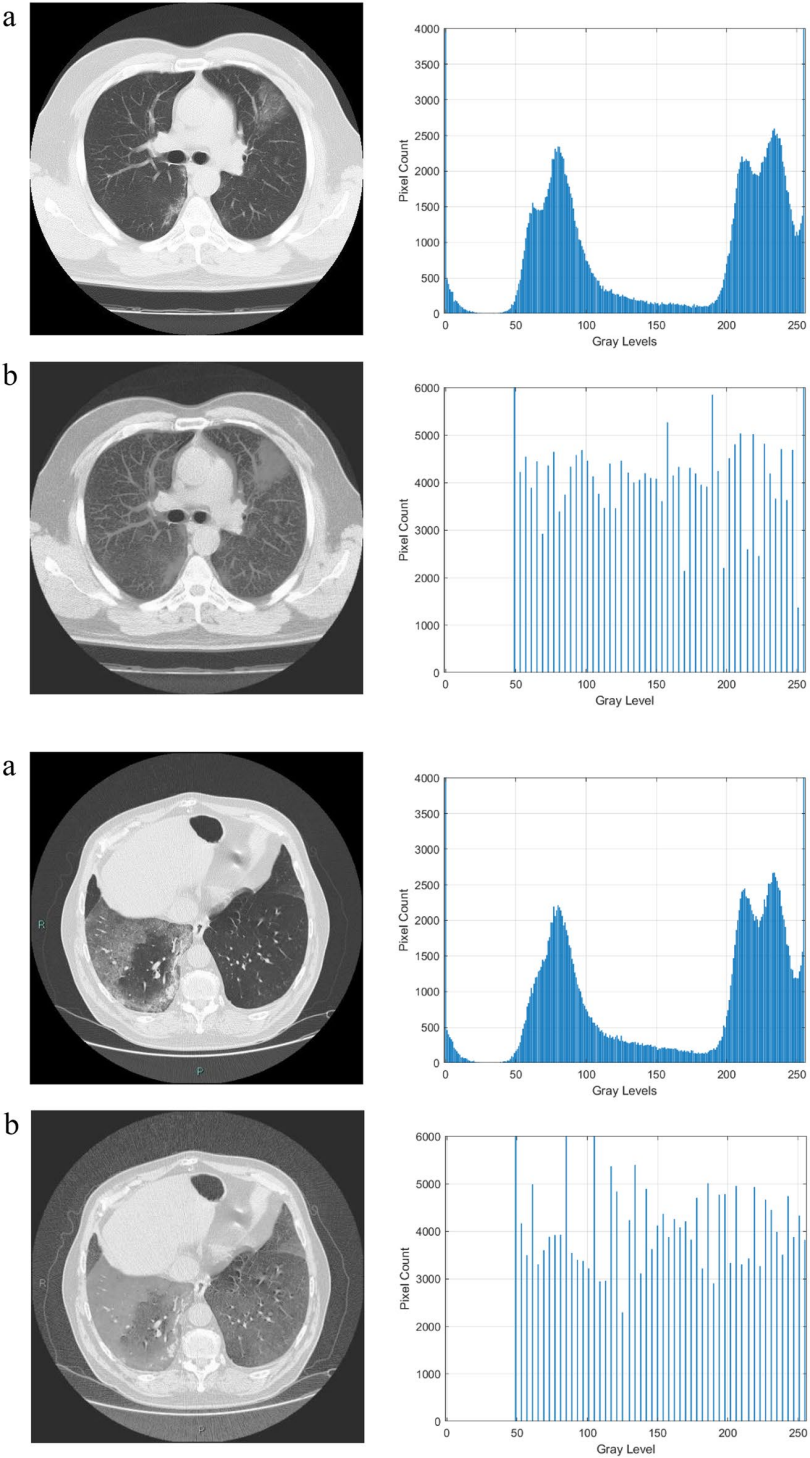

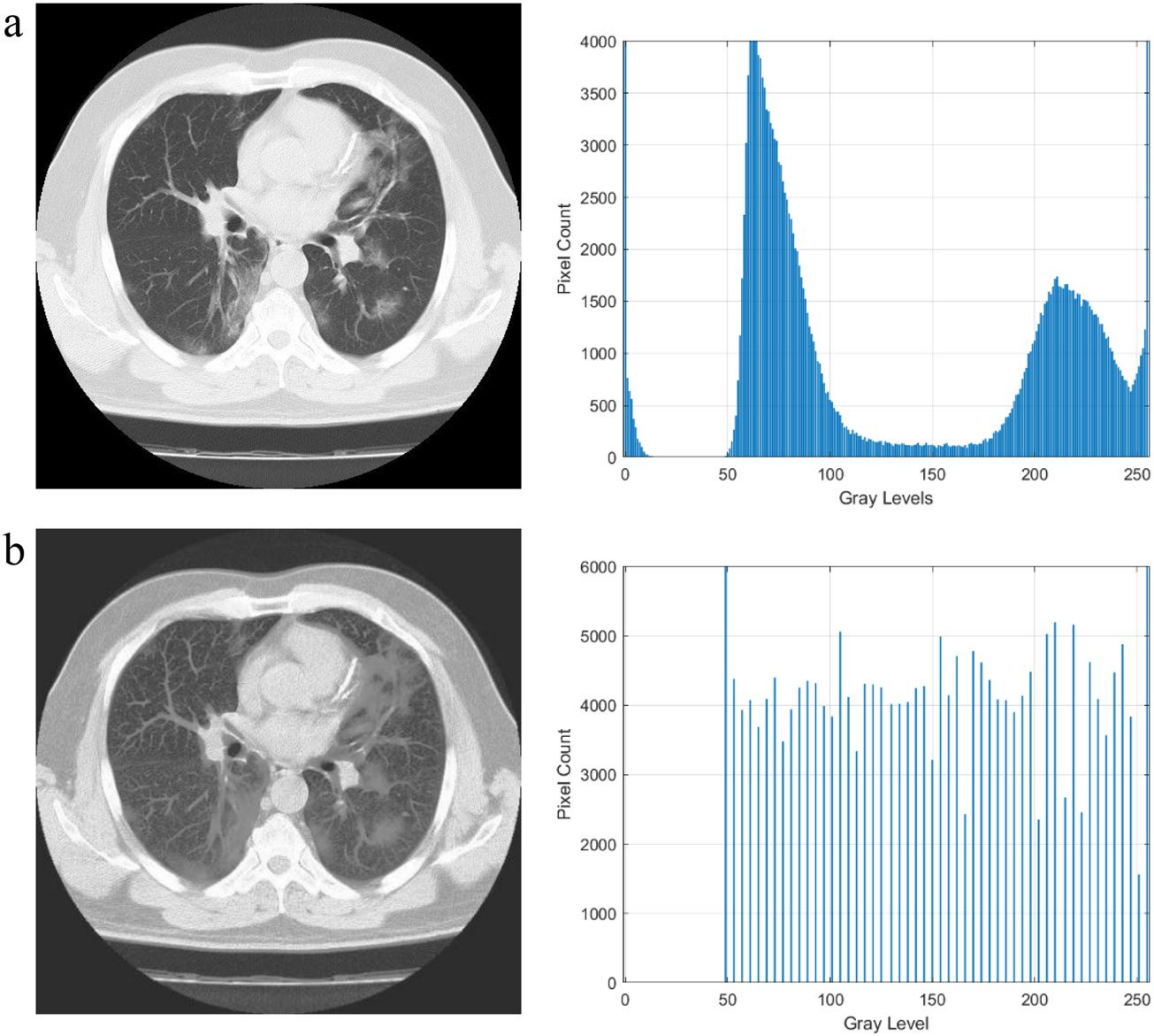
Results of the CT scan image enhancement with histogram analysis, (**a**) is the original image, (**b**) is the enhanced image.

**Figure 4:**
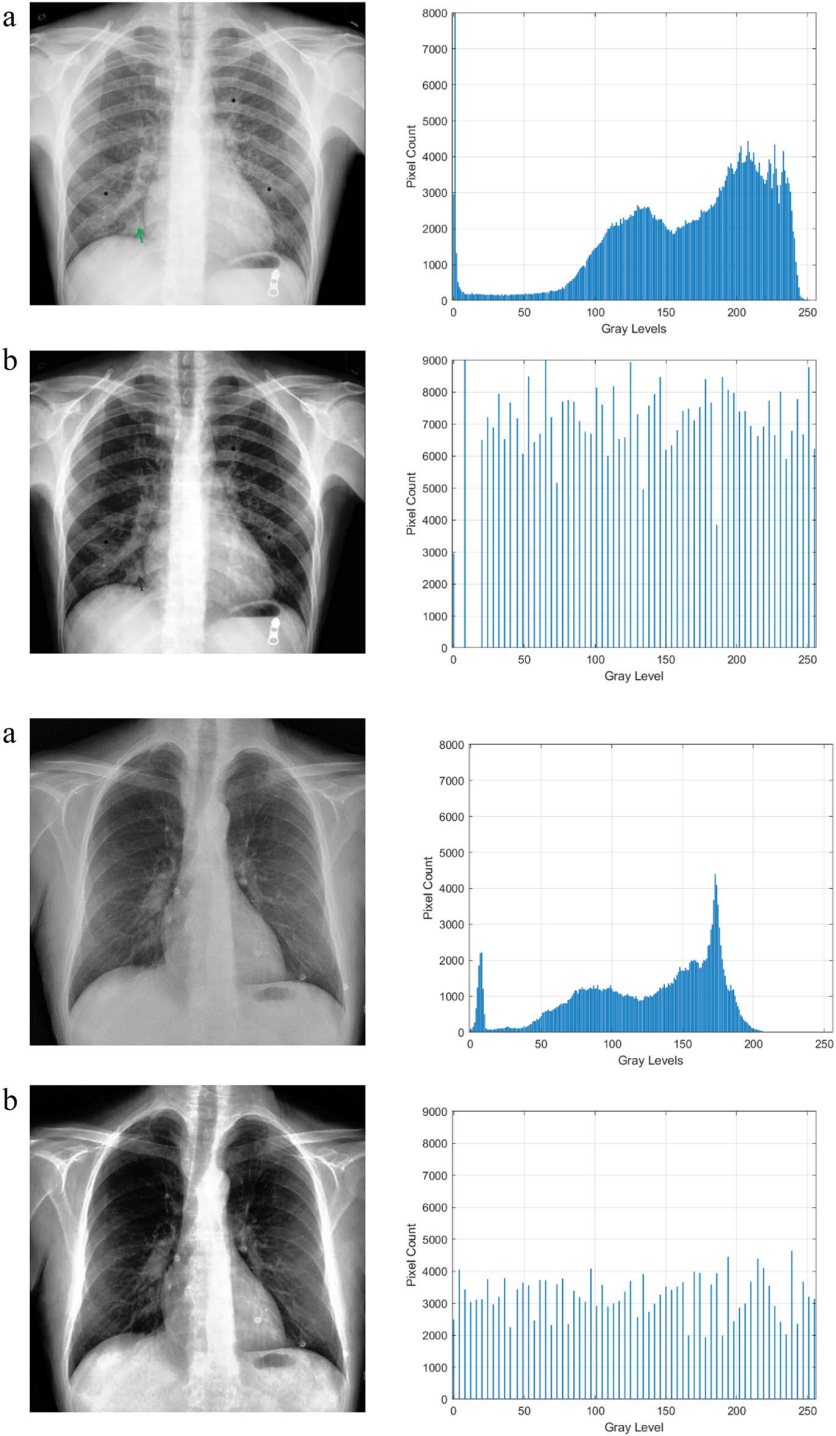

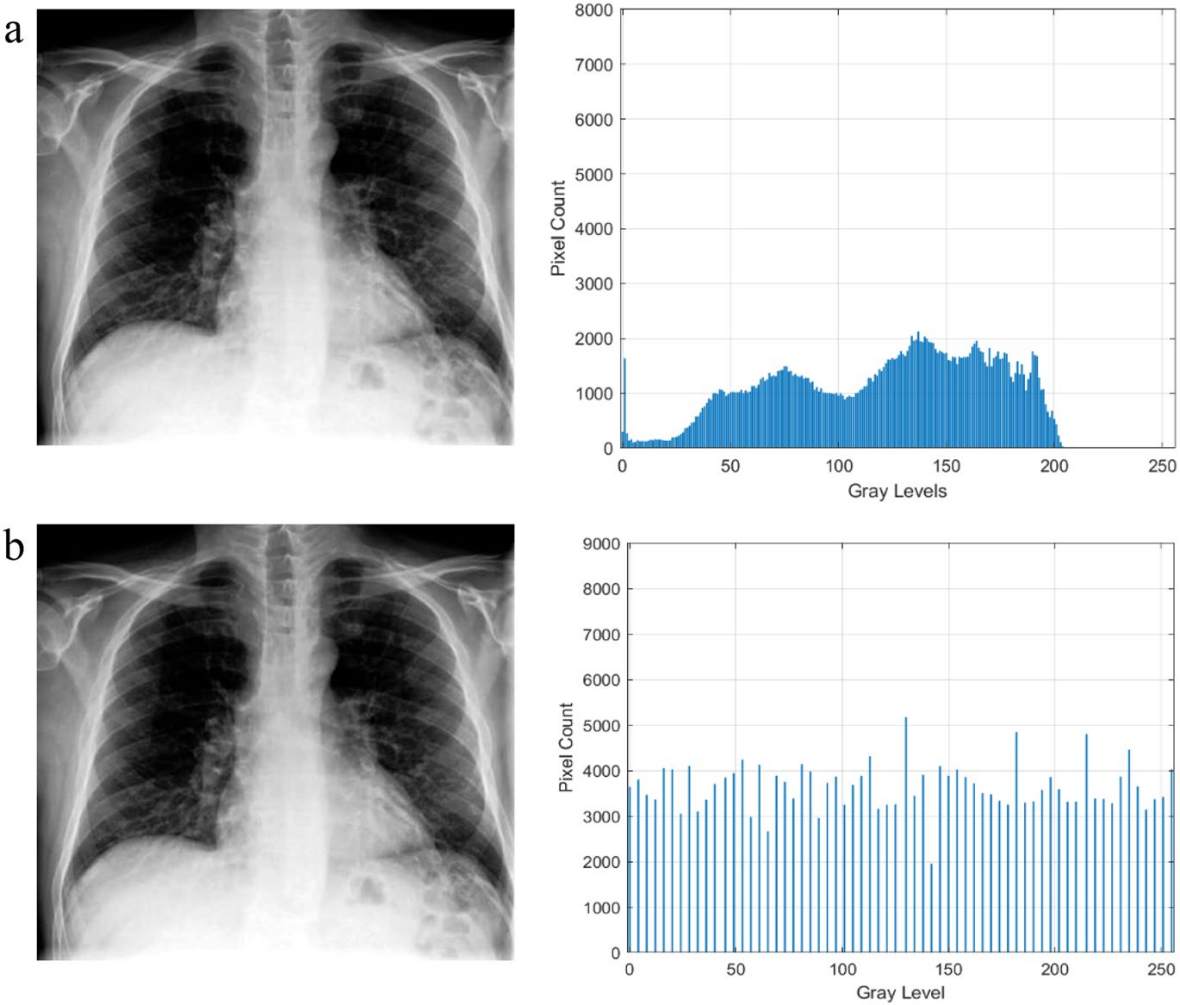
Results of the X-Ray image enhancement with histogram analysis, (**a**) is the original image, (**b**) is the enhanced image.

The qualitative outcomes of this model are shown in Figs. 3 and 4 respectively in CT scan and X-Ray images. The input images, enhanced images, and histogram plots are all shown in the figures. The original image pixel probability histogram plot appears dense, whereas the enhanced image pixel probability histogram plot appears scattered which indicates the improvement in the image’s contrast.

The histogram plots in Figs. 3 and 4 are used to quantitatively assess the impact of the proposed image enhancement method on the image characteristics. The histogram analysis reveals the differences between the input and improved image characteristics. Input images clearly show the loss of image details, but as can be seen from a histogram plot analysis, the contrast of the source images is stretched when compared to the suggested enhancement method. After the process of enhancement, areas that lack details also become brighter and more distinct. While the pixel intensity distribution in the enhanced image is more evenly distributed, the histogram plots demonstrate how compact the pixel probability distribution is in images. This demonstrated the improvements to the enhanced image contrast.

### Deep learning classification models

The objective of the proposed method is to effectively classify lung diseases into three categories using deep learning CNN methods. The DL technique has proven to be an essential tool in a variety of applications. CNN is a part of the DL family, which has attracted many researchers recently. The DL CNN performs well in medical imaging like MR, CT, and X-Ray classification^24^. Extracting the major features of the images is an important aspect of the image classification process in the model. These features are extracted by CNNs using filters in the convolutional layers.

### Proposed customized *CNN*

**Figure 5:**
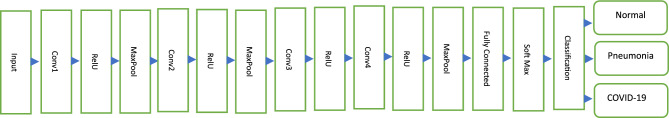
The proposed, customized CNN architecture.

The proposed CNN model is built from scratch to classify lung diseases in X-Ray and CT scans. The proposed customized CNN model has 4 “convolution layers”, 3 “pooling layers”, and the “fully connected layer”. In the training process, the input image size for the proposed customized CNN is 227 × 227. The “batch Normalization Layer”, “rectified linear layer” (ReLU layer) and “maxpooling” comes after the “convolutional layers” (ConvLs) as shown in Fig. 5.

In the CNN model, the batch normalization layer is used for stabilizing the learning and is applied right before the ReLU, while the pooling layer is used to reduce feature size extracted by the convolutional layers. The ‘fully connected’ and ‘softmax layers’ are used for lung diseases. The learnable parameters of the proposed customized CNN model are illustrated in Table 1.

**Table 1:** The learnable parameters for the customized CNN model.

Layer	Weight	Filters
Conv1	5 × 5 × 3 × 16	1 × 1 × 16
Conv2	5 × 5 × 16 × 32	1 × 1 × 32
Conv3	5 × 5 × 32 × 64	1 × 1 × 64
Conv4	5 × 5 × 64 × 128	1 × 1 × 128
Fully connected		3 × 1

In the context of CNNs, the numbers provided in Conv1 (5 × 5 × 3 × 16) are typically related to the shape and properties of a convolutional filter or kernel. 5 × 5: This refers to the spatial dimensions of the filter. A 5 × 5 filter will look at 5 pixels in height and 5 pixels in width at a time as it slides over the input image. 3: This is the depth (or channels) of the input to the convolutional layer which is the colored image with channels (Red, Green, Blue). However, as you progress deeper into a CNN, the depth of the layers can increase as they start to represent more abstract features. A depth of 3 suggests that the layer immediately preceding this one has 3 feature maps (or channels). 16: This indicates the number of filters (or kernels) of the given 5 × 5 size. Each filter will learn to detect different features in the input data. After the convolution operation, this would produce 16 separate feature maps. The depth of the output from this convolutional layer would be 16.

### Training plan

The input size for all images has been changed to 227 × 227, and the batch size has been set to 32. For validation, the accuracy was computed after every epoch. The optimal hyperparameters and settings of proposed customized CNN model are trained with learning rate = 0.0001, batch size 32, MaxEpochs = 30, iteration per epoch = 2, maximum number of iterations = 60, Momentum = 0.9, ValidationFrequency = 30. In order to achieve a stable activation value distribution throughout the training process, a batch normalization layer supports every convolutional layer. The batch normalization layer is typically used before the non-linearity layer, also known as ReLU. To prevent over-fitting, the early stopping strategy is also used. This method minimizes the possibility of the network overfitting on the training set by stopping the training process as soon as it notices no change in the validation loss value. The datasets were split into 30% for testing and 70% for training. To minimize classification bias and enhance training performance, a five-fold cross-validation technique was employed to identify the three lung diseases, with an equal number of observations maintained for each of the three classes.

**Figure 6:**
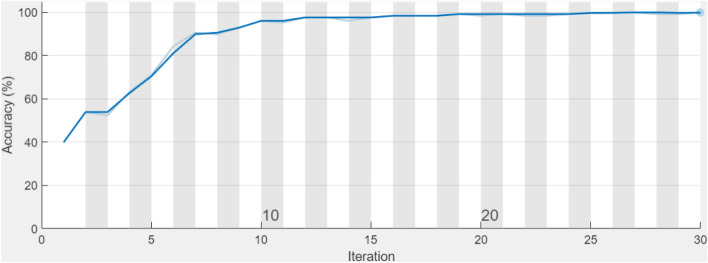
The proposed customized CNN model’s training process.

The training process along with the number of iterations for proposed CNN in CT scans is illustrated in Fig. 6. In the first 10 iterations of Fig. 6, there is considerable instability, but after 25 iterations, about 100 percent training accuracy is obtained. The proposed customized CNN model achieved the maximum training accuracy in CT scans, demonstrating how the proposed CNN's basic structure may reduce the number of layers and produce significantly more accurate results than transfer learning.

### Pre-trained CNNs

The CNN medical image training involves many images, which is challenging to achieve under regular testing conditions. Transfer learning (TL) is an alternative to this, which involves reusing CNNs that have been taught on large datasets. The TL is currently trending in research because it can be used to train CNNs with a relatively small dataset. The main advantages of the TL are saving training time, and better performance with small datasets. In this study, the two fine-tuned trained CNNs models are ‘Alex Net, and VGG16Net’ have been applied. These two models are already trained on a huge ImageNet dataset which is considered the “source dataset”. The fine-tuned CNN is using the pre-trained networks to train the “target datasets”, which are the CT scans, and X-Ray datasets. In the training process, the input image size for AlexNet is 227 × 227 pixels, and the input image size for VGG16Net is 224 × 224.

The AlexNet has five deep ConvLs. The input layer is of size 227 × 227 × 3. Three max-pool layers with 3 × 3 pool size. Three ‘fully connected layers’ arenamed FC6, FC7 and FC8. Finally, the ‘Softmax’ is the last layer that is used for the prediction of classes. While the VGG16Net has 16 deep ConvLs.

The input layer of VGG16 Net ‘Visual Geometry Group’ is of size 224 × 224 × 3. Five max-pool layers with 2 × 2 pool size. Three ‘fully connected layers’ named FC6, FC7 and FC8, and the final layer is the “Softmax” which is used for the prediction of classes. A few modifications were made to fine-tune the pre-trained CNN models by replacing the final layers, i.e., the “fully connected layer”, “softmax”, and “classification layer”. These three layers were replaced to classify the new target classes, i.e., COVID-19, Pneumonia and Normal.

The AlexNet and VGG16Net were trained and validated using five-fold cross-validation. During training, the optimizer uses stochastic gradient descent with momentum (SGDM) to reduce the loss function at each iteration across the whole training dataset.

**Figure 7:**
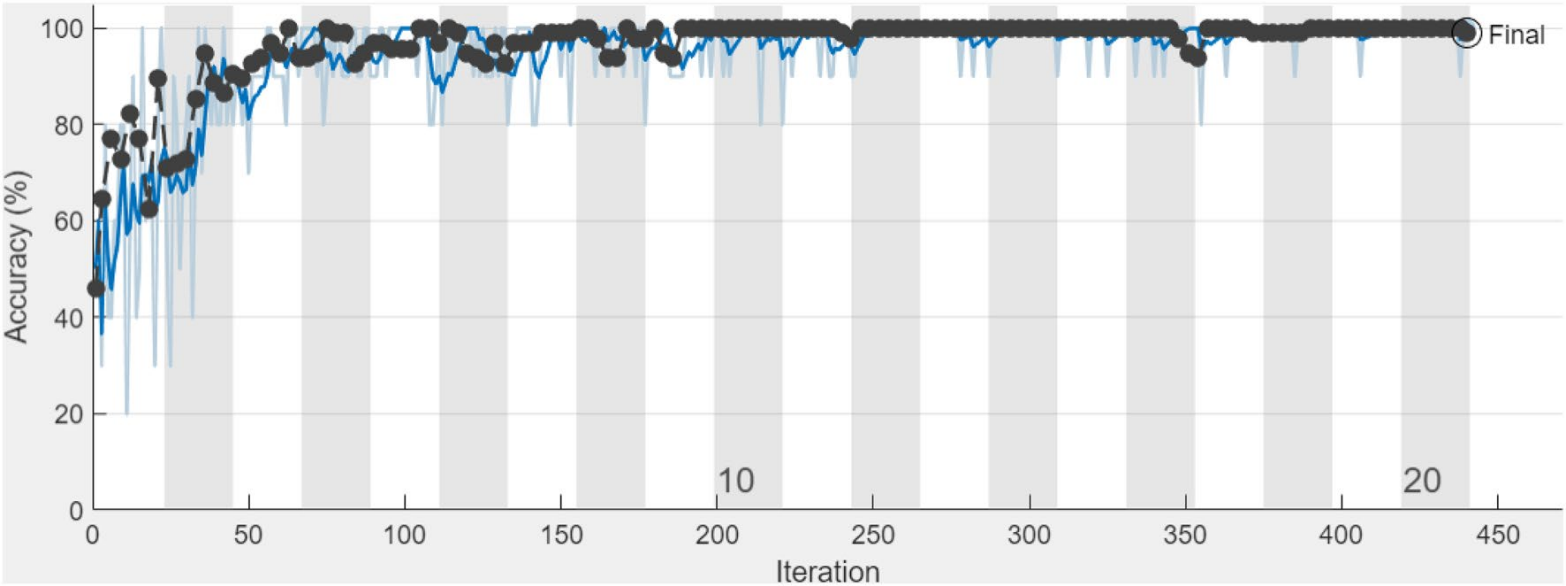
The process of fine-tuning the trained AlexNet.

**Figure 8:**
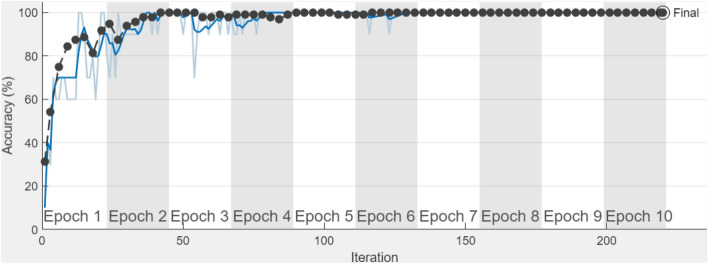
The process of fine-tuning the trained VGG16Net.

The optimal hyperparameters that were obtained for the training of trained CNNs AlexNet, and VGG16Net models are Training mode: Stochastic Gradient Descent, Mini-batch size = 10, trained with learning rate = 0.0001, Momentum = 0.9, MaxEpochs = 10, and the ValidationFrequency = 30. The two selected fine-tuned trained CNNs “AlexNet, VGG16Net” models have been modified in such a way that the weights of their last layers of “AlexNet, and VGG16Net” have been removed and re-trained using both X-Ray and CT scans datasets. The training process with the number of iterations for fine-tuned CNNs “AlexNet, VGG16” models are illustrated in Fig. 7, and Fig. 8 respectively.

The illustrated graphs indicate the ability of the fine-tuned trained CNNs “AlexNet, VGG16Net” models for lung disease classification. It can be seen from Figs. 7 and 8 that almost 100% accuracy is achieved after 300 iterations for fine-tuned trained AlexNe, and 100% accuracy is obtained after 150 iterations for fine-tuned trained VGG16Net.

### Image datasets

We used the following publicly available datasets, which comprise 1714 images of X-Ray and CT scans.

1.The CT scans dataset from the “Italian Society of Medical and Interventional Radiology (SIRM)^25^ and the Radiopaedia^26^ contain 337 COVID-19, 196 Pneumonia, and 277 Normal CT scans (Total of 810 images) in PNG format.

2. The X-Ray dataset from^27^ contains 237 of COVID-19, 250 of Pneumonia, and 417 of Normal Chest X-Rays (Total 904 images) in JPG format.

### Performance metrics

To assess the proposed image classification approach, the following measures are used: TP: “true positive” (TP), FP: “false positive”, TN: “true negative”, and FN: “false negative”. The classification measures that were used are as follows:

5 $$Accuracy = \frac{TP + TN}{{TP + TN + FP + FN}}$$

6 $$Sensitivity \left( {Recall} \right) = \frac{TP }{{TP + FN }}$$

7 $$Specificity = \frac{TN }{{TN + FP }}$$

8 $$Precision = \frac{TP }{{TP + FP }}$$

9 $$F1{\text{-}}Score = 2 \times \frac{{Precision~ \times ~Recall~}}{{Precision + Recall~}}$$

## Results and discussion

MATLAB R2021b on Windows 10 with processor Intel(R) Core i7 @ 2.80GHz with 16 GB RAM was used to produce the test results. Tests were carried out on a set of datasets that were divided into 70% training and 30% testing. The test set is a different set of data used to verify the model after it has been trained. Furthermore, the fivefold cross-validation is used to identify the three lung diseases. Table 2 illustrates the findings of plain X-Ray and CT scans (without image enhancement) for the three classes studied, which averaged about 96%.

**Table 2:** Obtained results of X-Ray and CT scans without image enhancement.

Method	Image dataset	Accuracy %	Sensitivity (Recall) %	Specificity %	Precision %	F1-Score %
Proposed customized CNN	X-Ray	96.40	95.30	96.30	98.30	96.77
	CT	96.60	96.50	96.30	96.10	96.30
Pre-trained VGG16Net	X-Ray	95.10	95.60	95.50	95.10	95.34
	CT scans	95.30	95.30	96.40	95.00	95.14
Pre-trained AexNet	X-Ray	96.40	96.20	95.60	95.70	96.00
	CT scans	96.70	96.20	96.10	95.80	96.00

The performance metrics for classification were enhanced by 3% after applying the proposed image enhancement method as a pre-processing step. As shown in Table 3, all performance metrics improved by more than 98% when using the proposed image enhancement model.

**Table 3:** Evaluation of proposed CNN and the fine-tuning trained models with preprocessing using proposed image enhancement.

Method	Image dataset	Accuracy %	Sensitivity %	Specificity %	Precision %	F1-Score %
Proposed customized CNN	X-Ray	98.60	98.40	98.50	98.30	98.35
	CT	98.80	98.50	98.40	98.60	98.55
Pre-trained VGG16Net	X-Ray	98.20	98.30	98.30	98.15	98.22
	CT scans	98.10	98.10	98.20	98.00	98.05
Pre-trained AlexNet	X-Ray	98.20	98.20	98.30	98.10	98.15
	CT scans	98.40	98.10	98.20	98.00	98.05

The deep learning classification models have been used in this work to classify-three classes of COVID-19, pneumonia, and normal using customized CNN and two pre-trained CNN models Alex Net, VGG16Net with a new image enhancement model proposed. The Accuracy, Sensitivity, and Specificity of the proposed deep learning models were all tested. The findings after using the proposed image enhancement showed that the customized CNN provided satisfactory classification performance with 96.80% accuracy in CT scans and 98.60% in X-Ray images, as shown in Table 3. The pre-trained VGG16Net can identify various chest disorders with an accuracy, of 98.20 on X-Ray images, and 98.10% on CT scans. As well as the pre-trained AlexNet can identify chest disorders with an accuracy, of 98.20 on X-Ray images, and 98.40% on CT scans. The fine-tuned trained CNNs “AlexNet, VGG16Net” succeeded to obtain good classification accuracy for the lung disease classification which reflects the benefits of applying the deep fine-tuned models for detection CT scans and in X-Ray images.

### Ablation study

The suggested structure of customized CNN is examined and proven to be valid using the ablation experiment concept. The goal of an ablation study is to understand the impact of removing or modifying specific elements of the model on its performance. Three experiments were used in an ablation study to examine the effects of varying the number of kernel filters on the performance of the proposed network. These subsets are (16, 16, 32 and 64), (16, 32, 64 and 64), (16, 32, 32 and 128) and (16, 32, 64 and 128). Table 4 provides an overview of the ablated models' performance.

**Table 4:** The ablation study of proposed customized CNN on CT images.

Experiment	Kernel filter (Convolutional Layer 1, 2, 3,4)	Accuracy %
Ablation Study 1	16, 16, 32 and 64	92.10
Ablation Study 2	16, 32, 64 and 64	92.50
Ablation Study 3	16, 32, 32 and 128	93.70
Proposed Mode	16, 32, 64 and 128	98.80

### Comparative analysis

The comparative results of proposed customized CNN model and the fine-tuned trained CNNs ‘AlexNet, VGG16Net’ models with other lung disease classification models are illustrated in Table 5. In this comparison, Irmak^28^, used two proposed CNNs for lung disease detection in X-Ray images. The achieved accuracy was 98.27%. Similarly, Hasan et al.^1^, presented a combination of deep features for classification CT scans with three lung disease cases. The maximum achieved accuracy was 97.50%. Moreover, Ucar and Korkmaz et al.^29^ proposed a new deep Bayes-Squeeze Net for the diagnosis of COVID-19. This model achieved 97.93% of classification X-Ray images of three lung disease conditions. While Wang et al.^30^, the proposed COVID-19 CT image classification achieved detection accuracy of up to 89.50% for binary classification. The proposed COVID-19 classification model from chest X-Ray images by Wang et al.^31^achieved 92.64% classification accuracy. Another approach was proposed by Nour et al.^32^ for the diagnosis of COVID-19 using deep features with Bayesian optimization. The achieved classification accuracy was 98.97% for X-Ray images. An integrated deep learning model for COVID-19 detection using both chest X-Ray and CT images was proposed by Roy^33^. Various transfer learning techniques have been used in this proposed model to extract features and determine which ones work best together. The experimental findings demonstrated that the ResNet50 with capsule network as an extractor-classifier pair with the combined dataset exhibits the best performance. With X-Ray and CT images, the proposed model achieves accuracy of 98.2% and 97.8%, respectively. Moreover, Song et al.^34^ proposed a deep CNN for the diagnosis of COVID-19 with CT scans. Their model achieved 96.93% classification accuracy. A densely attention mechanism-based network (DAM-Net) was proposed by Zahid et al.^35^ for the automatic detection of COVID-19 in CXRs. The DAM-Net was used to accurately identify COVID-19 by capturing image features. The performance was enhanced using DenseNet. The COVIDx data, which are available to the public, were used to evaluate this model. Likewise, Elmehdi^36^ used various CNNs (Resnet-18, InceptionV3, and MobileNetV2) to compare the effectiveness of different chest imaging techniques in the diagnosis of COVID-19 infection. The ResNet-18 was found to have the best overall precision and sensitivity, with values of 98.5% and 98.6%, respectively. With the same CNN models, CT scans have demonstrated superior performance when detecting positive cases when compared to CXR images.

**Table 5:** The comparison outcomes using state-of-the-art methods.

Method	Images dataset	Accuracy %	Sensitivity (Recall)%	Specificity %	Precision %
Irmak^28^, 2020	X-Ray	98.27	98.09	99.13	98.49
Ucar et al.^29^, 2020	X-Ray	98.30	–	99.10	–
Wang et al.^31^, 2020	X-Ray	93.40	93.30	95.76	–
Nour et al.^32^, 2020	X-Ray	98.97	89.39	99.75	–
Hasan et al.^1^, 2020	CT	97.50	96.80	98.10	98.31
Wang et al.^30^, 2021	CT	89.50	87.00	88.00	–
Song et al.^34^, 2021	CT	93.00	93.00	93.00	93.00
Roy, and Das^33^, 2023	CT	98.20	–	–	–
Roy and Das^33^, 2023	X-Ray	97.80	–	–	–
Zahid et al.^35^, 2023	X-Ray	97.22	96.87	99.12	95.54
Elmehdi et al.^36^, 2023	CT	96.50	97.20	97.40	97.50
Elmehdi et al.^36^, 2023	X-Ray	87.70	92.30	88.80	93.40
Proposed method	X-Ray	98.60	98.40	98.50	98.30
Proposed method	CT	98.80	98.50	98.40	98.60

Table 5 provides a brief comparison of the work presented in the literature and the proposed deep learning customized CNN. This comparison runs in two different directions. The first direction is based on the works that have been evaluated using X-Ray images. In this direction, the proposed deep learning customized CNN attained an accuracy of 98.60%, while Nour et al.^30^ attained higher classification accuracy of 98.97%. Nour et al.^32^ CNN model was applied as a deep feature extractor to feed the support vector machine (SVM) and decision tree. In addition, the Bayesian optimization algorithm was used to optimize the hyperparameters of the machine learning models, which made it slightly more accurate at classifying images than the proposed deep learning customized CNN. Moreover, another comparison is to be made between the proposed deep learning customized CNN and the earlier research that was evaluated on CT images. When compared on CT scans to all the previously described models, the proposed customized CNN model has the highest average classification accuracy of 98.80%. The tested deep learning models' improved classification accuracy may help medical staff diagnose chest disorders from X-Ray and CT scan images, improving patient outcomes and potentially saving lives.

## Conclusions

This study presents deep learning models for image classification. A new image enhancement algorithm was developed in the pre-processing step using a new k-symbol Lerch transcendent functions for accurate classification performance. The deep learning classification was carried out by the proposed customized CNN model as well as by two fine-tuned trained CNNs models “AlexNet, and VGG16Net”. Furthermore, the images used in this study were obtained from two publicly available datasets. When tested with two publicly available datasets, the proposed customized CNN model classification model achieved classification accuracy, sensitivity, and specificity of 98.60%, 98.40%, and 98.50% for the X-Ray image dataset respectively, with 98.80%, 98.50%, and 98.40% for CT scans dataset. The achieved results reflect the strengths of the image enhancement model as a pre-processing step. Moreover, the detection results of the fine-tuned trained CNNs “AlexNet, and VGG16Net” succeeded to obtain a good enough classification rate for lung diseases from chest X-Ray and CT images. The class imbalance of lung diseases, caused by an abundance of images from one class and a dearth of images from another, may have limited the study's ability to accurately identify the diseases. Possible future improvements include adding more fine-tuned trained CNNs with the capability to classify more infections from balance of lung diseases images while keeping the same level of effectiveness.

## Data Availability

The datasets analyzed during this study are the standard lung diseases and are available from^25–27^.
